# Supplementary material for: Diffusion-mediated HEI10 coarsening can explain meiotic crossover positioning in Arabidopsis
Source: Nat Commun. 2021 Aug 3;12:4674. doi: 10.1038/s41467-021-24827-w (PMC8333306; doi:10.1038/s41467-021-24827-w)
Supplement: Supplementary file 1 — Supplementary Information [file 41467_2021_24827_MOESM1_ESM.pdf]

Diffusion-mediated HEI10 coarsening can explain meiotic crossover positioning in *Arabidopsis*

Morgan *et al.*

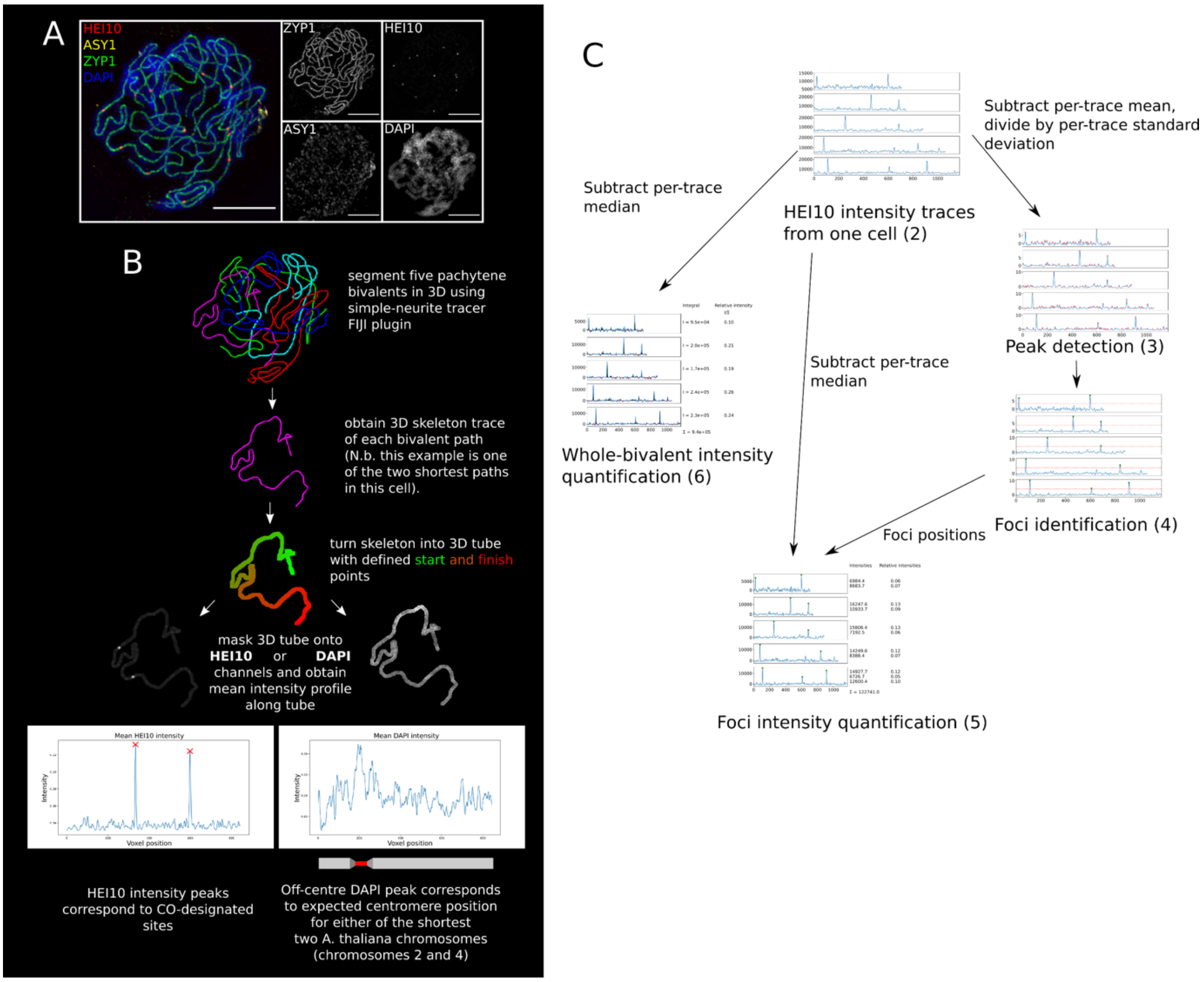

Supplementary Fig. 1. 3D-SIM image analysis pipeline. a, Maximum intensity projection of a wild-type Col-0 late-pachytene cell stained for HEI10, ASY1, ZYP1 and DAPI. Late-HEI10 foci mark crossover sites, ZYP1 highlights the synaptonemal complex, DAPI binds to DNA and ASY1 localises to any regions of asynapsis that are present in the cell. Representative image shown. Data was collected from 520 wild-type late-pachytene bivalents for further analysis. Scale bars = 5 µm. **b,** Synapsed chromosomal paths can be accurately defined in 3-dimensions by tracing along the ZYP1 and ASY1 channels using the simple neurite tracer (SNT) plugin to FIJI. This generates a 3D-skeleton path along each synapsed pair of chromosomes. A 3D-rendering of the five synapsed chromosomal paths present in this cell, generated using SNT, is shown, with each path highlighted in a different colour **(b,** top). Each path is arranged into a linear array of voxels with defined start and finish points. Spheres are placed at each voxel to generate a 3D tube. This 3D-tube can then be masked onto the HEI10 or DAPI channels of the original 3D-SIM image z-stack. The mean voxel intensity for each voxel can then be measured from start to finish along the tube to either map the position of HEI10 foci or to define the position of the centromere along each synapsed pair of chromosomes (DAPI). However, as the DAPI signal was much noisier than the HEI10 signal, it was not always possible to accurately define the position of a single centromere-associated DAPI intensity peak for each chromosome. Nevertheless, in the example shown here, there is a clear off-centre DAPI peak consistent with the acrocentric centromere position on either of the two shortest *A. thaliana* chromosomes. **c,** Workflow for late-HEI10 foci identification and intensity quantification. HEI10 intensity profiles along five bivalent traces from one example cell are shown. The numbered steps in this workflow correspond to the numbered steps in the Image Analysis section of the Methods.

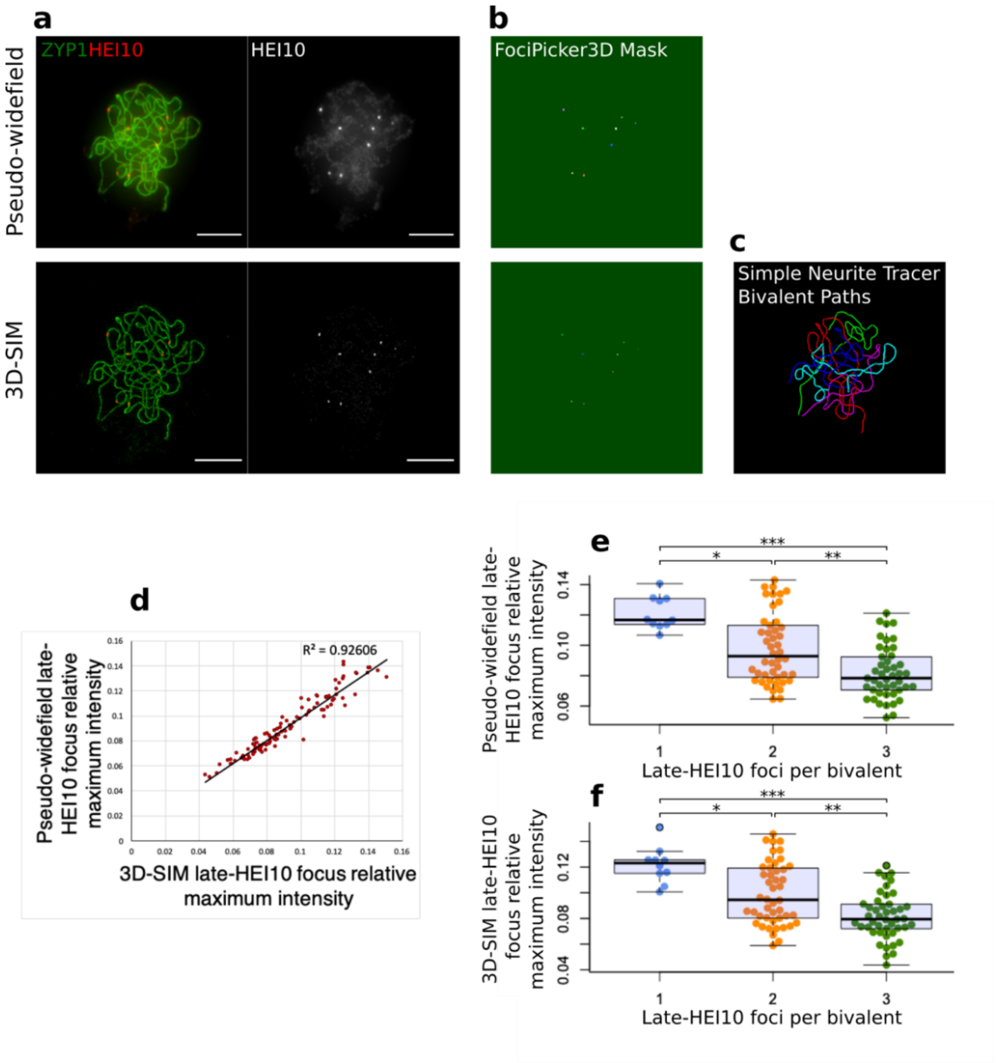

Supplementary Fig. 2. Measuring late-HEI10 foci intensity in 3D-SIM and pseudo-widefield images using FociPicker3D. a, Maximum intensity projections of pseudo-widefield (top) and 3D-SIM (bottom) images, generated from the same raw imaging data of the same wild-type late-pachytene cell labelled for ZYP1 (green) and HEI10 (red). Images of the HEI10 only channel are also shown. Representative images are shown. Data was collected from 10 late-pachytene cells for further analysis. Scale bars = 5 µm. **b**, Maximum intensity projections of image masks generated by the FociPicker3D FIJI plugin, using a uniform background threshold of 15000 and 30000 and minimum pixel size of 10 and 5 for the pseudo-widefield and 3D-SIM HEI10 images, respectively. Different late-HEI10 foci that are identified using this method are highlighted in different colours. **c,** The paths of all five bivalents can be identified and traced in 3-dimensions (each bivalent is highlighted in a different colour in the 3D-rendering) from the 3D-SIM ZYP1 channel imaging data using the Simple Neurite Tracer FIJI plugin. **d**, Scatter plot showing the relative maximum intensity values (normalized against the summed maximum intensity of all late-HEI10 foci within the same cell) of the same late-HEI10 foci, identified using FociPicker3D, obtained from pseudo-widefield vs 3D-SIM images, generated from the same raw imaging data, in a subset of 10 high-quality wild-type cells. The fitted linear regression and R-squared value is shown. n=109 foci. **e, f,** Plots showing the relative maximum intensity of foci on bivalents with a total of 1 (n=10 foci), 2 (n=46 foci) or 3 (n=45 foci) foci per bivalent obtained from pseudo-widefield (**e**) or 3D-SIM (**f**) images of the same raw data from the same subset of 10 wild-type late-pachytene cells. Boxplots show: center line, median; box limits, upper and lower quartiles; whiskers, 1.5x interquartile range. Bonferroni corrected p-values from a one-sided Dunn Test for multiple comparisons are shown, ***$p < 0.0005$, **$p<0.005$, *$p<0.05$. Exact *p*-values are as follows: Pseudo-widefield 1 vs 2 foci ($Z = 2.77$, p unadjusted = 0.0055, p adjusted = 0.017), 1 vs 3 foci (Z = 4.79, p unadjusted = 0.0000016, p adjusted = 0.0000049), 2 vs 3 foci (Z = 3.38, p unadjusted = 0.00073, p adjusted = 0.0022), 3D-SIM 1 vs 2 foci ($Z = 2.57$, p unadjusted = 0.010, p adjusted = 0.031), 1 vs 3 foci (Z = 4.59, p unadjusted = 0.0000044, p adjusted = 0.000013), 2 vs 3 foci ($Z = 3.39$, p unadjusted = 0.00071, p adjusted = 0.0021). Source data are provided as a Source Data file.

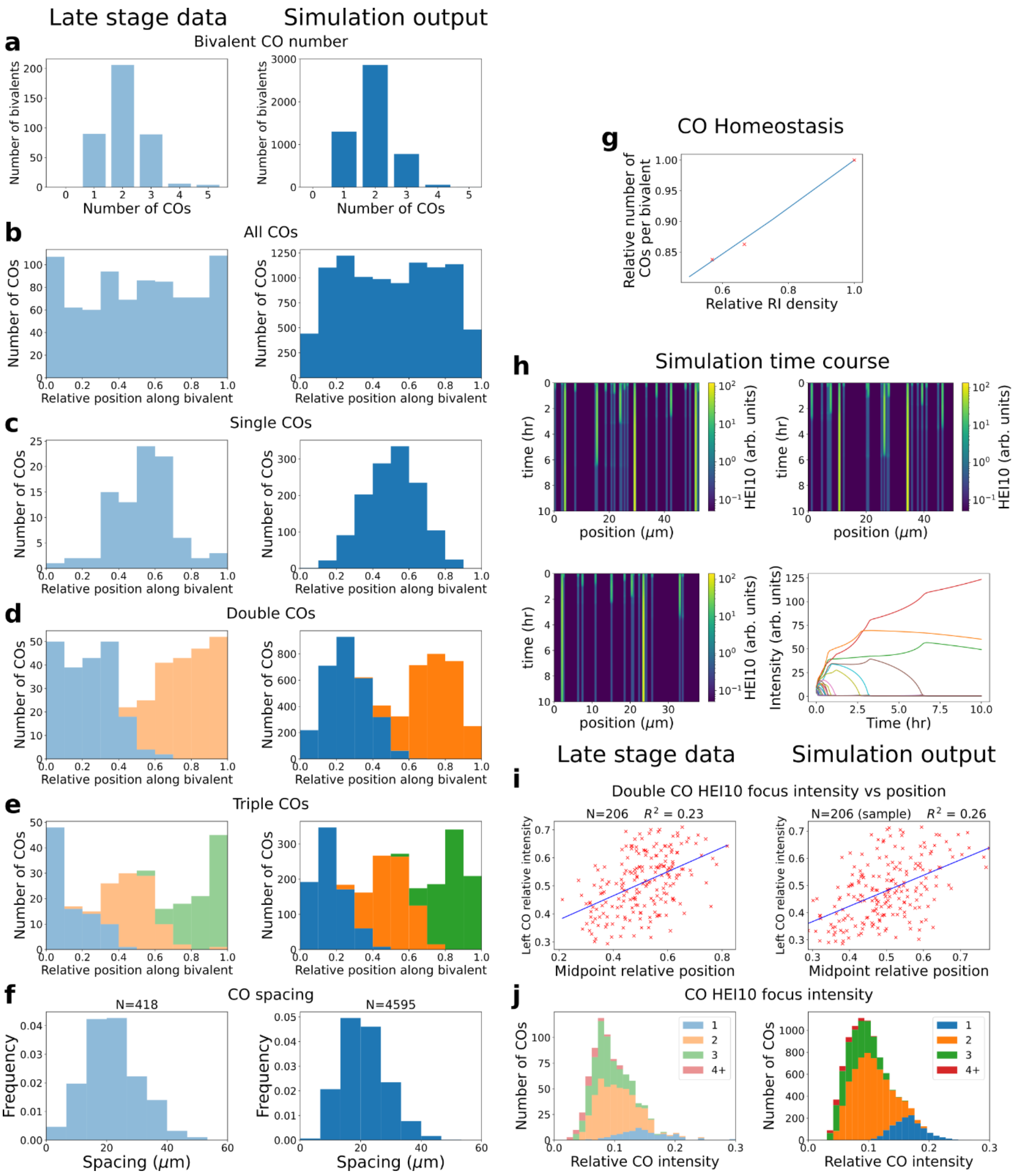

Supplementary Fig. 3. Model simulation results without elevated HEI10 loading close to chromosome ends. **a-f,** Comparison of cytology data from late-stage bivalents (left) with simulation outputs (right). Cytology data is the same as in Fig. 2, replotted here to aid comparison. Crossovers (COs) in the experimental data are defined as the positions of late-HEI10 foci. **a,** Number of COs per bivalent. **b-e,** CO positions along bivalents. **b,** All COs. **c,** Bivalents with single CO. **d,** Stacked histograms for double COs, showing positions of 1st/2nd CO. **e,** Triple COs, showing positions of 1st/2nd/3rd CO. **f,** Distribution of spacing between adjacent COs. **g,** Effect of varying initial RI density on CO number per bivalent relative to wild-type (data [1]: red crosses; model prediction: blue line). **h,** Example simulation kymographs of HEI10 foci intensities in cases resulting in triple (top left), double (top right), single (bottom left) COs. Shading indicates log_e of foci intensities. Traces of foci intensity against time *(*bottom right*)*, showing the same simulation as in the triple CO case (top left). **i,** Analysis of double CO HEI10 focus intensities; experiments (left), simulations (right): On arbitrarily aligned SC, intensity of left CO, relative to sum of both intensities, against relative position (measured from left end) of the midpoint between the two COs (CO 'catchment area'). Blue lines: linear-regression best-fits. **j,** Histograms of intensities of CO HEI10 foci, relative to sum of all foci within a cell, colour-grouped according to CO number per bivalent (inset); experiments (left), simulations (right). Source data are provided as a Source Data file.

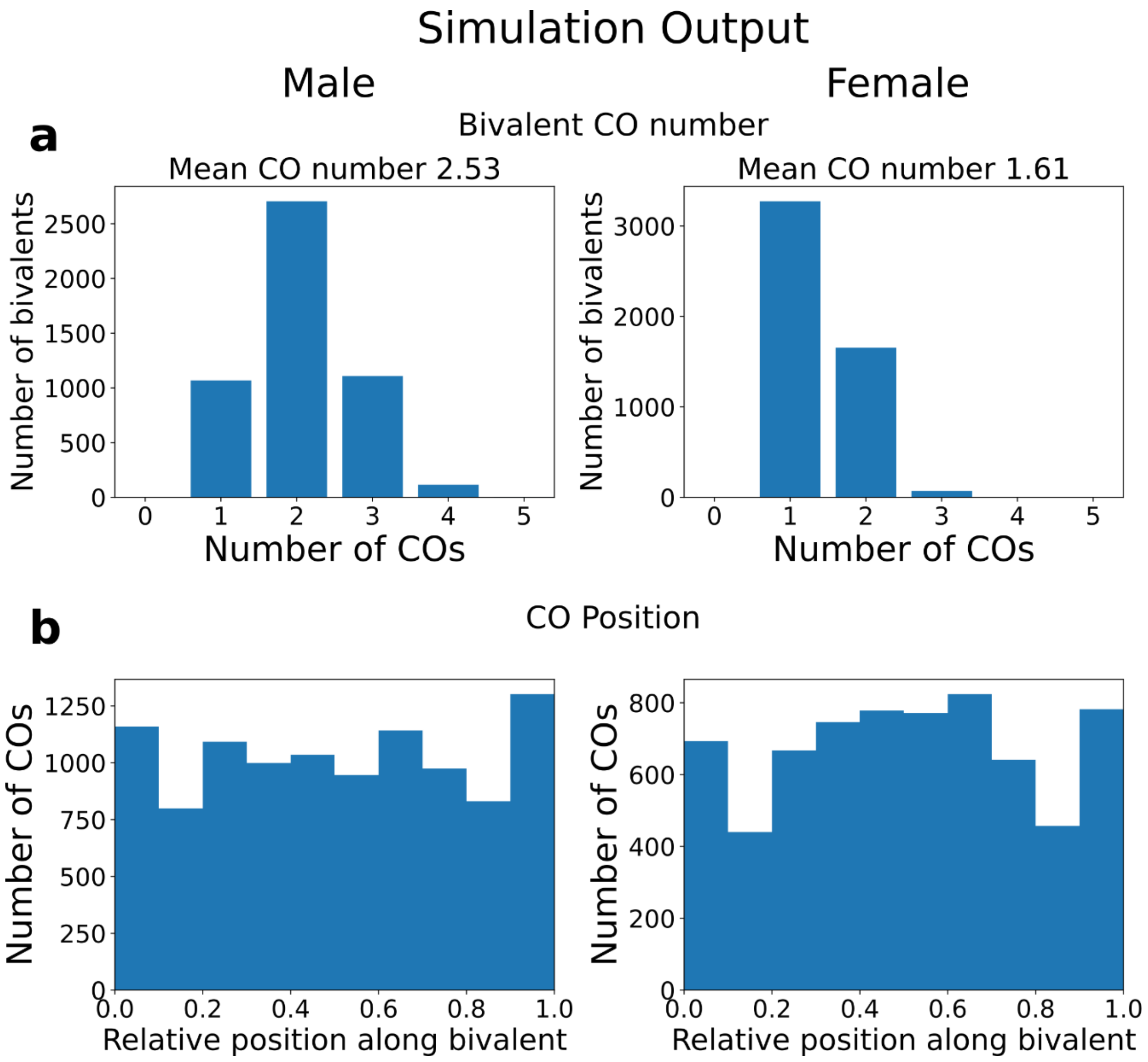

Supplementary Fig. 4. Comparison of model simulation predictions for male and female meiocytes. Left: simulated male bivalents (same simulation data as in Fig. 2); Right: simulated female bivalents. **a,** Number of COs per bivalent. **b,** CO positions along bivalents for all COs. Source data are provided as a Source Data file.

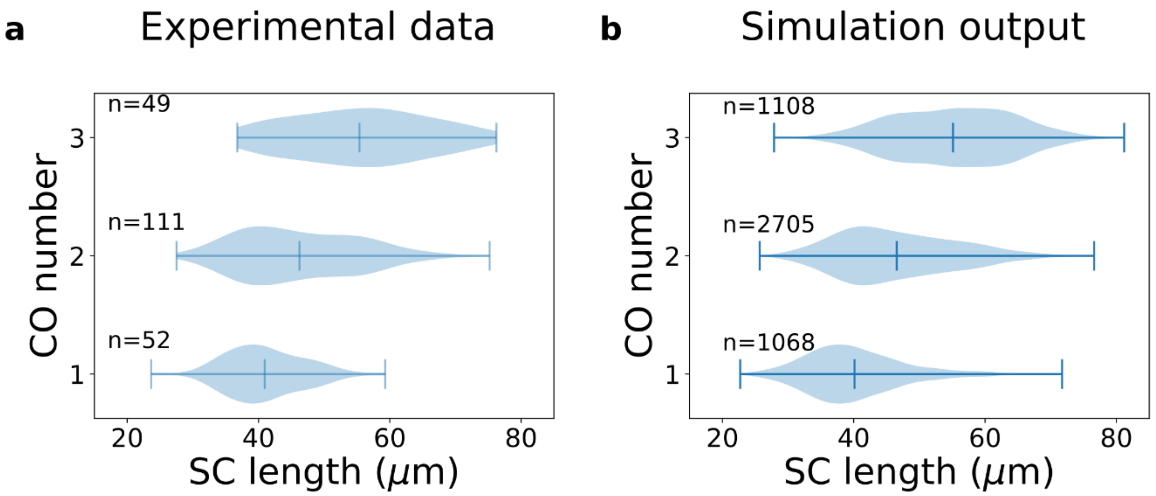

Supplementary Fig. 5. Crossover number against bivalent SC length. Violin plots of bivalent SC lengths, grouped according to number of COs (as defined by the position of late-HEI10 foci in the experimental data). Data are shown for bivalents with 1, 2 or 3 COs, which make up the bulk of the experimental data (96%, 212/220 bivalents) and simulation data (98%, 4881/5000 bivalents). Blue vertical lines show minimum, mean and maximum of data in each class. **a,** Wild-type experimental data. **b,** Output for WT simulations (same parameters as in Fig. 2). Wild type data from 49 cells from 9 plants. Source data are provided as a Source Data file.

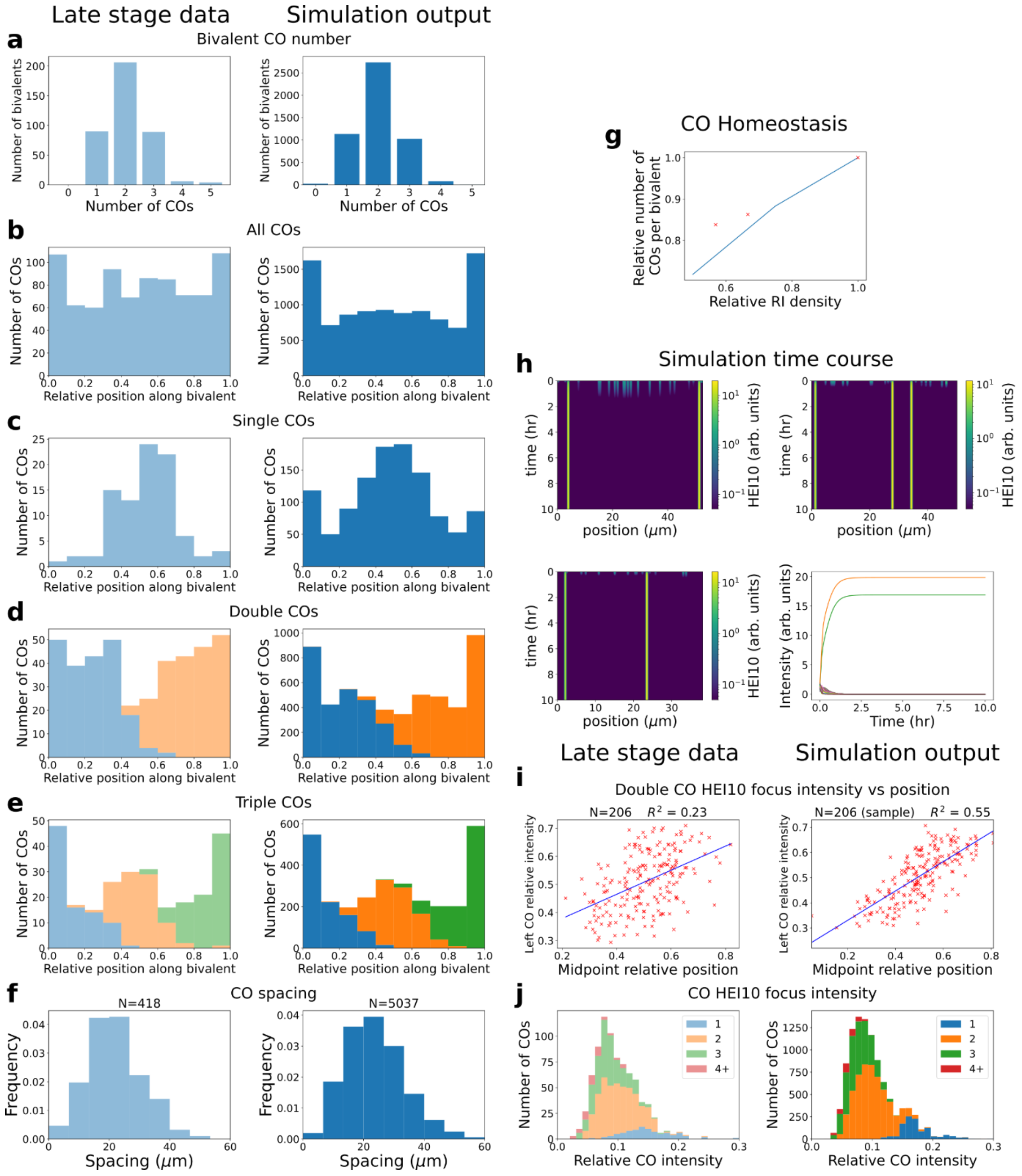

Supplementary Fig. 6. Model simulation results with modified RI escape rate $\beta(C) = \beta_C / \left(1 + \delta e^{C/K_C}\right)$.

a-f, Comparison of cytology data from late-stage bivalents (*left*) with simulation outputs (*right*). Cytology data is the same as in Fig. 2, replotted here to aid comparison. Crossovers (COs) in the experimental data are defined as the positions of late-HEI10 foci. **a,** Number of COs per bivalent. **b-e,** CO positions along bivalents. **b,** All COs. **c,** Bivalents with single CO. **d,** Stacked histograms for double COs, showing positions of 1st/2nd CO. **e,** Triple COs, showing positions of 1st/2nd/3rd CO. **f,** Distribution of spacing between adjacent COs. **g,** Effect of varying initial RI density on CO number per bivalent relative to wild-type (data [1]: red crosses; model prediction: blue line). **h,** Example simulation kymographs of HEI10 foci intensities in cases resulting in triple (top left), double (top right), single (bottom left) COs. Shading indicates $\log_e$ of foci intensities. Traces of foci intensity against time (bottom right), showing the same simulation as in the triple CO case (top left). **i,** Analysis of double CO HEI10 focus intensities; experiments (left), simulations (right): On arbitrarily aligned SC, intensity of left CO, relative to sum of both intensities, against relative position (measured from left end) of the midpoint between the two COs (CO 'catchment area'). Blue lines: linear-regression best-fits. **j,** Histograms of intensities of CO HEI10 foci, relative to sum of all foci within a cell, colour-grouped according to CO number per bivalent (inset); experiments (left), simulations (right). All parameters are the same as in Fig. 2, except that initial HEI10 loading

has been reduced by a factor of close to 0.14 ($C_0 = 0.93\ au$, $\sigma = 0.30\ au$, $c_0 = 0.16\ au\ \mu m^{-1}$), and the new parameter is $\delta = 1.25$. Source data are provided as a Source Data file.

Supplementary Table 1. A list of genotyping primers used in this study.

Primer name	Sequence
hei10-2-F	5'-AAGGAGTTCCCAGAGATGCTC-3'
hei10-2-R	5'-GCCAGCAAGACAGAACAGTTC-3'
LBb1.3	5'-ATTTTGCCGATTTCGGAAC-3'
HEI10-F	5'-TATTTCTGTGTCTTGAGCTTAGTGC-3'
HEI10-R	5'-CAGTCACGACGTTGTAAAACGACG-3'

Supplementary Table 2. SC lengths used for simulations.

Chromosome Index	Length (µm)	Standard deviation (µm)
1	37.5	4.93
2	40.4	5.06
3	45.7	5.86
4	53.8	7.18
5	59.8	7.13

Means and standard deviations of the normal distributions used for generating male SC lengths in the simulations, for each of the 5 chromosomes, ordered shortest to longest. These statistics were extracted from the late-stage WT data, for each cell (n=74) in which all SCs were traced successfully, as described in the Methods section. For female meiosis, both the mean and standard deviation were taken to be 60% of the values given here.

Supplementary reference

1. Xue, M. *et al.* The number of meiotic double-strand breaks influences crossover distribution in Arabidopsis. *Plant Cell* **30**, 2628 (2018).
