# Supplementary material for: Diffusion-mediated HEI10 coarsening can explain meiotic crossover positioning in Arabidopsis
Source: Nat Commun. 2021 Aug 3;12:4674. doi: 10.1038/s41467-021-24827-w (PMC8333306; doi:10.1038/s41467-021-24827-w)
Supplement: Supplementary file 3 — Reporting Summary [file 41467_2021_24827_MOESM3_ESM.pdf]

nature research

Corresponding author(s): Prof. Martin Howard

Last updated by author(s): Oct 7, 2020

Reporting Summary

Nature Research wishes to improve the reproducibility of the work that we publish. This form provides structure for consistency and transparency in reporting. For further information on Nature Research policies, see our Editorial Policies and the Editorial Policy Checklist.

Statistics

For all statistical analyses, confirm that the following items are present in the figure legend, table legend, main text, or Methods section.

n/a	Confirmed	
[ ]	[x]	The exact sample size (*n*) for each experimental group/condition, given as a discrete number and unit of measurement
[ ]	[x]	A statement on whether measurements were taken from distinct samples or whether the same sample was measured repeatedly
[ ]	[x]	The statistical test(s) used AND whether they are one- or two-sided<br>*Only common tests should be described solely by name; describe more complex techniques in the Methods section.*
[ ]	[x]	A description of all covariates tested
[ ]	[x]	A description of any assumptions or corrections, such as tests of normality and adjustment for multiple comparisons
[ ]	[x]	A full description of the statistical parameters including central tendency (e.g. means) or other basic estimates (e.g. regression coefficient) AND variation (e.g. standard deviation) or associated estimates of uncertainty (e.g. confidence intervals)
[ ]	[x]	For null hypothesis testing, the test statistic (e.g. *F*, *t*, *r*) with confidence intervals, effect sizes, degrees of freedom and *P* value noted<br>*Give P values as exact values whenever suitable.*
[x]	[ ]	For Bayesian analysis, information on the choice of priors and Markov chain Monte Carlo settings
[x]	[ ]	For hierarchical and complex designs, identification of the appropriate level for tests and full reporting of outcomes
[x]	[ ]	Estimates of effect sizes (e.g. Cohen's *d*, Pearson's *r*), indicating how they were calculated

Our web collection on statistics for biologists contains articles on many of the points above.

Software and code

Policy information about availability of computer code

Data collection	3D-SIM images were acquired on a Zeiss Elyra PS1 microscope utilising Zeiss' ZEN Black software (14.0.17.201). Bivalent skeleton traces were obtained using the Simple Neurite Tracer FIJI (2.1.0/1.53c) plugin. 3D-intensity traces were generated using custom Python scripts [https://github.com/jfozard/HEI10].
Data analysis	Python 3.7.6 ; using scipy 1.4.1, numpy 1.18.1, imageio 2.6.1, pandas 1.0.1, statsmodels 0.11.0 and matplotlib 3.3.2. Preliminary data analysis and data entry using Excel (16.16.27_201012 Microsoft). Simulations performed using JuliaPro 1.4.0, using DifferentialEquations.j. 6.11.0.<br>R 4.0.03; using packages 'kruskal.test' and 'dunn.test'

For manuscripts utilizing custom algorithms or software that are central to the research but not yet described in published literature, software must be made available to editors and reviewers. We strongly encourage code deposition in a community repository (e.g. GitHub). See the Nature Research guidelines for submitting code & software for further information.

Data

Policy information about availability of data

All manuscripts must include a data availability statement. This statement should provide the following information, where applicable:

- Accession codes, unique identifiers, or web links for publicly available datasets
- A list of figures that have associated raw data
- A description of any restrictions on data availability

Imaging data used in this study, which is associated with all figures, was deposited to the Image Data Resource (https://idr.openmicroscopy.org) under accession number idr0107 (https://doi.org/10.17867/10000162).

Field-specific reporting

Please select the one below that is the best fit for your research. If you are not sure, read the appropriate sections before making your selection.

☒ Life sciences ☐ Behavioural & social sciences ☐ Ecological, evolutionary & environmental sciences

For a reference copy of the document with all sections, see nature.com/documents/nr-reporting-summary-flat.pdf

Life sciences study design

All studies must disclose on these points even when the disclosure is negative.

Sample size

No sample size calculations were performed as part of this study. Sample sizes were chosen based on the amount of high-quality imaging data that could be experimentally obtained. Primary HEI10 focal positioning and intensity data was collected from all genotypes until data had been obtained from at least 3 plants of each genotype and until sufficient data had been collected to allow meaningful comparisons to be drawn between experimental positioning data and simulation outputs.

Data exclusions

HEI10 intensity and positioning traces were excluded from bivalents where bivalent 3D skeleton traces were prematurely truncated during the image-analysis step. This occasionally occurred if branched segments were incorporated within the initial Simple Neurite Tracer bivalent traces, which were then incompatible with downstream image analysis steps. Cells that could not be clearly staged as early-, mid- or late-pachytene were also excluded from the analysis.

Replication

To ensure reproducibility, all HEI10 positioning and intensity data were obtained from all genotypes using the same computational analysis pipeline. This removed the potential experimenter bias that can occur as a consequence of scoring and measuring HEI10 focal positions manually. Data from this publication should, therefore, be readily reproducible using the primary imaging data and custom Python scripts that will be made available prior to publication.
To verify the reproducibility of the HEI10 focal intensity measurements and to ensure these results were not an artefact of our analysis pipeline, we also performed image analysis using two separate approaches and using both pseudo-widefield and 3D-SIM imaging data. Both approaches replicated the same significant negative relationship between HEI10 focal intensity and focus number per bivalent.

Randomization

Randomization was not relevant to this study. Plants were assigned to different groups based on their genotype.

Blinding

Investigators were not blinded to group allocation during the study. Blinding was not relevant to this study as HEI10 positioning and intensity data were obtained from all genotypes using the same computational image analysis pipeline,. The image analysis pipeline was optimised using the wild-type data and the HEI10 under- and over-expressor data was subsequently analysed using the exact same pipeline.

Reporting for specific materials, systems and methods

We require information from authors about some types of materials, experimental systems and methods used in many studies. Here, indicate whether each material, system or method listed is relevant to your study. If you are not sure if a list item applies to your research, read the appropriate section before selecting a response.

Materials & experimental systems

n/a	Involved in the study	
☐	☒	Antibodies
☒	☐	Eukaryotic cell lines
☒	☐	Palaeontology and archaeology
☒	☐	Animals and other organisms
☒	☐	Human research participants
☒	☐	Clinical data
☒	☐	Dual use research of concern

Methods

n/a	Involved in the study	
☒	☐	ChIP-seq
☒	☐	Flow cytometry
☒	☐	MRI-based neuroimaging

Antibodies

Antibodies used

For immunocytology, primary antibodies were supplied by Prof. F.C.H. Franklin (University of Birmingham). The following primary polyclonal antibodies were used at 1:500 dilutions: anti-HEI10 (rabbit), anti-ZYP1 (rat) and anti-ASY1 (guinea-pig). The following secondary polyclonal antibodies were used at 1:200 dilutions: anti-rat Alexa Fluor 488, anti-rabbit Alexa Fluor 555 and anti-guinea pig Alexa Fluor 647. Secondary antibodies were obtained from ThermoFisher
- Goat anti-Rat IgG (H+L) Cross-Adsorbed Secondary Antibody, Alexa Fluor 488 from Thermo Fisher Scientific, catalog # A-11006, RRID AB_2534074.
- Goat anti-Rabbit IgG (H+L) Cross-Adsorbed Secondary Antibody, Alexa Fluor 555 from Thermo Fisher Scientific, catalog # A-21428, RRID AB_2535849. Goat anti
- Guinea Pig IgG (H+L) Highly Cross-Adsorbed Secondary Antibody, Alexa Fluor 647 from Thermo Fisher Scientific, catalog # A-21450, RRID AB_2735091.

Validation

All primary antibodies used during this study have been widely used and validated as part of previous publications:
anti-ASY1: Armstrong, S. J., Caryl, A. P., Jones, G. H. & Franklin, F. C. H. Asy1, a protein required for meiotic chromosome synapsis, localizes to axis-associated chromatin in Arabidopsis and Brassica. Journal of Cell Science 115, 3645 (2002).
anti-ZYP1: Higgins, J. D., Sanchez-Moran, E., Armstrong, S. J., Jones, G. H. & Franklin, F. C. H. The Arabidopsis synaptonemal complex protein ZYP1 is required for chromosome synapsis and normal fidelity of crossing over. Genes & development 19, 2488–2500 (2005).
anti-HEI10: Lambing, C. et al. Arabidopsis PCH2 Mediates Meiotic Chromosome Remodeling and Maturation of Crossovers. PLOS Genetics 11, e1005372- (2015).
